# RETRACTION: Risk Factors of Foot Ulcers in Patients With End‐Stage Renal Disease on Dialysis: A Meta‐Analysis

**DOI:** 10.1111/iwj.70316

**Published:** 2025-03-06

## Abstract

**RETRACTION:** YanS. , YaoD. , WangY. , and ZhangJ. , “Risk Factors of Foot Ulcers in Patients With End‐Stage Renal Disease on Dialysis: A Meta‐Analysis,” International Wound Journal 21, no. 1 (2024): e14348, 10.1111/iwj.14348.37667546 PMC10782048

The above article, published online on 04 September 2023, in Wiley Online Library (http://onlinelibrary.wiley.com/), has been retracted by agreement between the journal Editor in Chief, Professor Keith Harding; and John Wiley & Sons Ltd. Following an investigation by the publisher, all parties have concluded that this article was accepted solely on the basis of a compromised peer review process. The editors have therefore decided to retract the article. The authors did not respond to our notice regarding the retraction.

**RETRACTION:** S. Yan , D. Yao , Y. Wang , and J. Zhang , “Risk Factors of Foot Ulcers in Patients With End‐Stage Renal Disease on Dialysis: A Meta‐Analysis,” International Wound Journal 21, no. 1 (2024): e14348, 10.1111/iwj.14348.37667546 PMC10782048

The above article, published online on 04 September 2023, in Wiley Online Library (http://onlinelibrary.wiley.com/), has been retracted by agreement between the journal Editor in Chief, Professor Keith Harding; and John Wiley & Sons Ltd. Following an investigation by the publisher, all parties have concluded that this article was accepted solely on the basis of a compromised peer review process. The editors have therefore decided to retract the article. The authors did not respond to our notice regarding the retraction.

